# Plant viruses induce plant volatiles that are detected by aphid parasitoids

**DOI:** 10.1038/s41598-023-35946-3

**Published:** 2023-05-30

**Authors:** Panagiotis G. Milonas, Eirini Anastasaki, Aikaterini Psoma, Georgios Partsinevelos, Georgios N. Fragkopoulos, Oxana Kektsidou, Nikon Vassilakos, Apostolos Kapranas

**Affiliations:** 1 Scientific Directorate of Entomology and Agricultural Zoology, Benaki Phytopathological Institute, 8 Stefanou Delta Street, 14561 Kifissia, Greece; 2 Scientific Directorate of Phytopahtology, Benaki Phytopathological Institute, 8 Stefanou Delta Street, 14561 Kifissia, Greece; 3 Laboratory of Applied Zoology and Parasitology, School of Agriculture, Aristotle University of Thessaloniki, 541 24 Thessaloníki, Greece

**Keywords:** Chemical ecology, Behavioural ecology

## Abstract

*Aphis gossypii* (Sternorrhyncha: Aphididae) aphids are vectors of important plant viruses among which cucumber mosaic virus (CMV) and potato virus Y (PVY). Virus-infected plants attract aphid vectors and affect their behavior and growth performance either positively or negatively depending on mode of transmission. Viruses cause changes in the composition and the amount of volatile organic compounds (VOCs) released by the plant that attract aphids. The aphid parasitoid *Aphidius colemani* (Hymenoptera: Aphelinidae) has been shown to have higher parasitism and survival rates on aphids fed on virus-infected than aphids fed on non-infected plants. We hypothesized that parasitoids distinguish virus-infected plants and are attracted to them regardless of the presence of their aphid hosts. Herein, we examined the attraction of the *A. colemani* parasitoid to infected pepper plants with each of CMV or PVY without the presence of aphids. The dynamic headspace technique was used to collect VOCs from non-infected and CMV or PVY-infected pepper plants. Identification was performed with gas chromatography-mass spectrometry (GC–MS). The response of the parasitoids on virus-infected vs non-infected pepper plants was tested by Y-tube olfactometer assays. The results revealed that parasitoids displayed a preference to CMV and PVY infected plants compared to those that were not infected.

## Introduction

Infection by a plant virus causes several phenotypic, physical and physiological changes in plants including alteration of color, size, texture, amino acids and phytohormone levels, distortion of cell structures, decrease of photosynthetic capacity in lower leaves and decrease of nutrient uptake resulting in delay of plant growth and development^1,2^ Some of the plant chemicals altered by plant viruses are carbohydrates and polyphenols, as well as enzymes involved in reactive oxygen species (ROS) production like peroxidase, catalase, ascorbate peroxidase, and superoxide dismutase^3^. Of particular interest is the manipulation of the plant defense pathways by the viruses during the infection process^4,5^. In several studies with insect transmitted viruses, an interference with the salicylic acid (SA) and jasmonic acid (JA) defense signaling was shown^6,7^.

These metabolic changes affect the biology of the insect vectors of the respective plant virus; life-cycle parameters of insect vectors, such as longevity, growth rate and fecundity differ on virus-infected plants compared to those on uninfected plants^8,9^. Particularly for aphid vectors, the mode of virus transmission is related to the observed performance of aphids on virus infected plants^10^. Moreover, they are attracted more to persistently transmitted (PT) viruses’ infected plants compared to non-infected plants^11,12^. Interestingly, aphid vectors were also found to be attracted to non-persistently transmitted (NPT) viruses’ infected plants despite their poor performance on these plants^5,13^. However, aphids’ performance on host plants infected by PT or NPT viruses is a species-specific trait. In certain cases, performance of aphid vectors on NPT infected host plant is improved^14^.

Insect herbivores are guided towards suitable oviposition and feeding sites through a variety of certain volatile organic compounds (VOCs) emitted by host plants^15^. Plant pathogens, including viruses, alter the volatile profile of their host plants which is utilized by foraging vectors for host location^11,16^.

Herbivory causes the induction of altered blend of volatiles emitted by plants (Herbivore Induced Plant Volatiles (HIPVs) that are used as synomones by parasitoids in order to locate their hosts^17–23^.

The interaction between parasitoids, their hosts and the plant species, is becoming more complex when plant pathogens are involved^24^. The role of pathogen induced volatiles in interactions between the pathogen and its insect vectors has been mainly focusing on the facilitation of spread of the pathogen towards other plants^13,25,26^. Although it is acknowledged that three-way interactions should be the focus in studying systems consisted of pathogens, plants and vectors, there is still little information about the effect of pathogens on the third trophic level^27,28^. Hence, there is increasing evidence that plant pathogens may also play a role in modifying natural enemy behavior. For instance, *Cotesia marginiventris* Cresson parasitoids were more attracted to fungus infested peanut plants attacked by *Spodoptera exigua* than to non-infested ones^29^. In another example the specialist parasitoid of *Diaphorina citri*, *Tamarixia radiata*, was attracted more toward *Candidatus* Liberibacter asiaticus (Las) -infected than uninfected citrus plants^30^. On the other hand, there are also cases where plant pathogens do not modify parasitoid behavior^31^. The differences recorded on the effect of pathogen infection towards the behavior of natural enemies of herbivores could be related to the presence or not of close evolutionary links within the system under study^32,33^.

Plant viruses also affect the performance of natural enemies. Mauck et al.^34^ found equal response of *A. colemani* parasitoids toward odors of aphid-infested control and CMV-infected *C. pepo* plants, however, parasitism was higher on aphids feeding on infected plants. Similarly, Joffrey et al.^28^ didn’t observe any difference in the preference of *A. colemani* parasitoids for turnip yellows virus (TuYV)-infected *Camelina sativa* plants, infested by aphids or not but parasitoid adults were smaller when emerged from viruliferous aphids compared to those emerged by non-viruliferous aphids. In these studies, the response of *A. colemani* adult parasitoids was examined either in the presence of aphids on the plants^34^ or in choice set-up without dynamic headspace VOCs movement and therefore other factors besides virus induced volatiles, such as visual stimuli or volatiles emitted by the herbivore itself were not excluded from the assays^28^. Aphid parasitoids respond to HIPVs as synomones for locating their aphid hosts^35–37^. Moreover, *A. colemani* respond to plant volatiles from un-infested plants to locate host's habitat^38^ and to HIPVs^39^. Recently it was found to respond and attracted to bacterial origin volatiles without the presence of aphids^40^. However, the olfactory response to virus infection without the presence of aphids has not been investigated.

We hypothesize that *A. colemani* adults are attracted to virus infected plants to secure location of host habitats. By responding to virus infected plants, parasitoids are likely to increase their successful foraging for aphid hosts for parasitisation^38^. Specifically, in the present study we test the hypothesis that *A. colemani* adult parasitoids discriminate volatiles from CMV or potato virus Y (PVY) infected pepper plants without the presence of aphids. Both CMV and PVY are common viruses associated with solanaceous plants including pepper^41^. For this purpose, we assess whether (i) infection by CMV or PVY induced changes in the VOCs profile of pepper plants and (ii) *A. colemani* adult parasitoids are attracted to CMV or PVY infected pepper plants without the presence of aphid-vectors.

## Results

### Response to olfactometer

**Figure 1:**
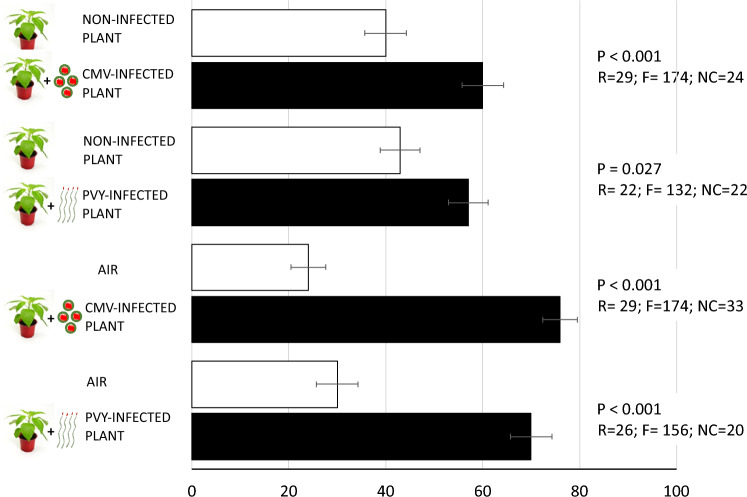
Percentage [mean + standard error (SE)] of female *A. colemani* wasps choosing volatiles emitted by virus-infected plants versus uninfected control plants or clean air in a Y-tube olfactometer. R = number of plants/replicates, F = number of females used, NC = no choice.

To test the attraction of adult parasitoids towards virus-infected pepper plants we performed pair-wise preference tests in which parasitoids were exposed to a combination of either virus infected plants and non-infected plants or virus-infected plants and clean air. There was no significant effect of the species of virus on the response of the parasitoids (GLM; χ^2^ = 0.0, df = 1, *P* = 1). Parasitoids were more attracted to CMV-infected pepper plants versus non-infected pepper plants (GLM; χ^2^ = 11.59, df = 1, *P* = 0.001) as well as to PVY-infected pepper plants versus non-infected pepper plants (GLM; χ^2^ = 4.88, df = 1, *P* = 0.027) (Fig. 1). Parasitoids were also more attracted to CMV-infected pepper plants versus clean air (GLM; χ^2^ = 69.66, df = 1, *P* < 0.001) as well as to PVY-infected pepper plants versus clean air (GLM; χ^2^ = 33.79, df = 1, *P* < 0.001) (Fig. 1).

### Headspace analysis of plant volatiles from virus infected plants

**Figure 2:**
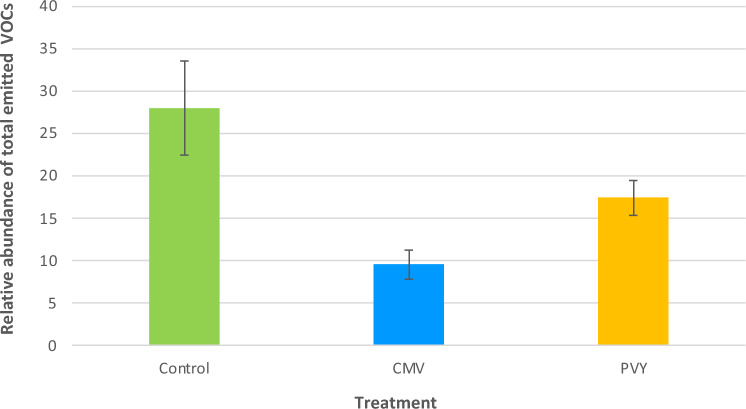
Total relative abundance of Volatile Organic Compounds (VOCs) emissions expressed as mean of the peak areas of the VOCs compared to the peak area of the internal standard ± SE. VOCs were collected by dynamic headspace trapping from non-infected (control), CMV-infected (CMV) and PVY-infected pepper plants.

**Figure 3:**
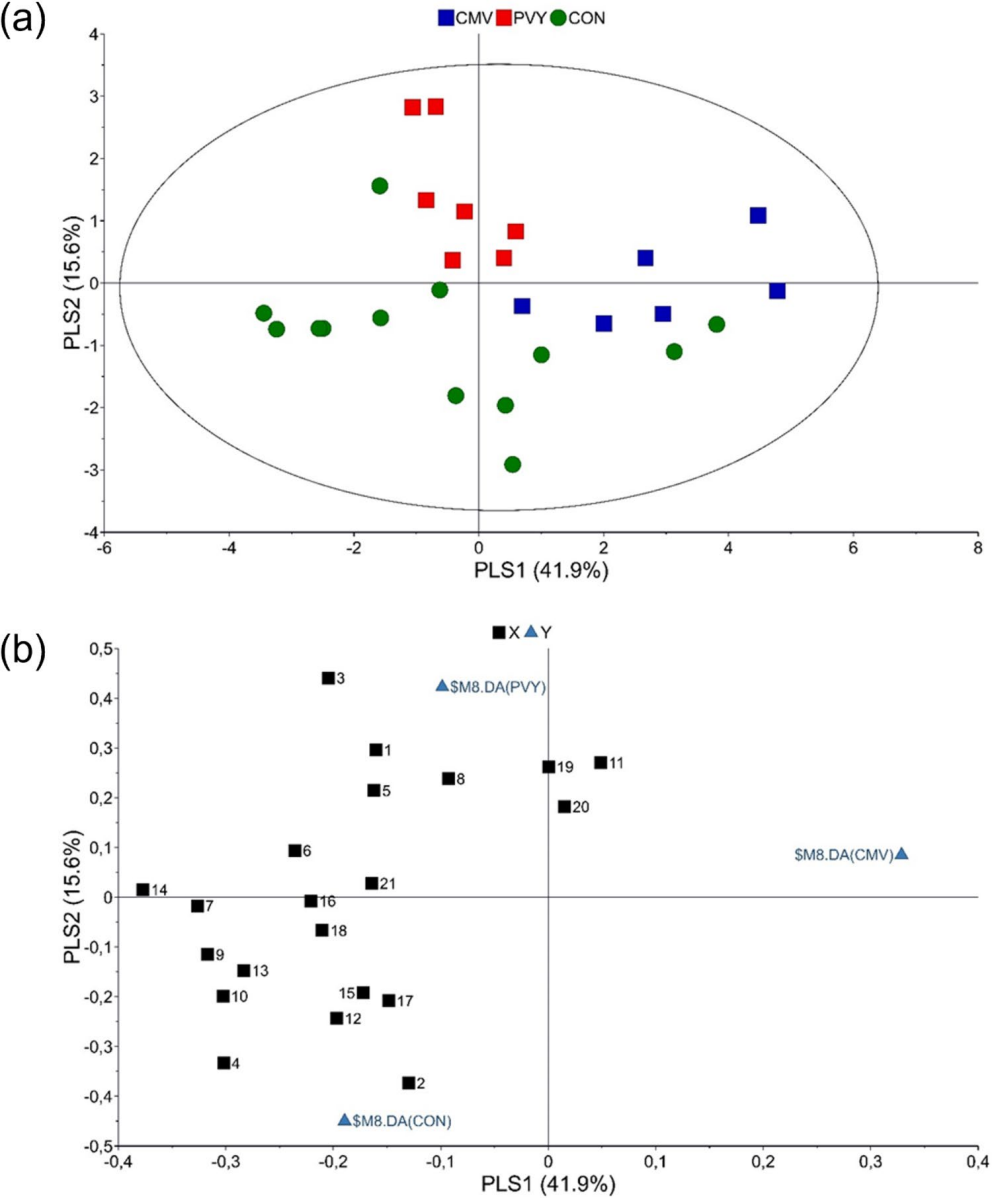
Projection Latent Structures Discriminant Analysis (PLS-DA) of the data of identified VOCs from non-infected (CON) and CMV or PVY infected pepper plants. (**a**) Score plot of the first two PLS components with explained variance in brackets. The ellipse defines Hotelling’s T2 confidence region (95%) and (**b**) Loading plot of the PLS-DA components that shows the contribution of each of the compounds to the first two principal components. For the interpretation of numbers refer to Table 1.

**Figure 4:**
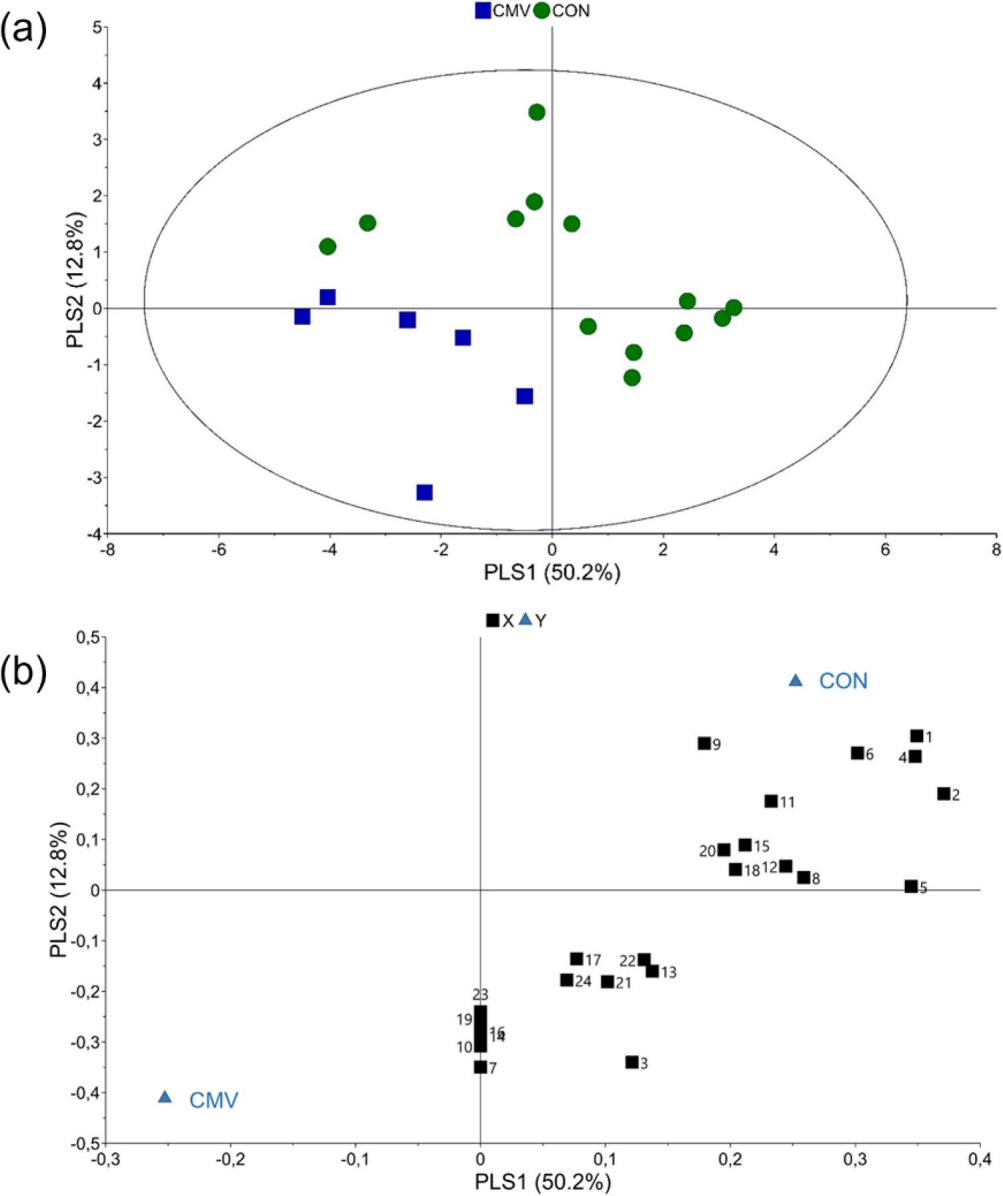
Projection Latent Structures Discriminant Analysis (PLS-DA) of the first two PLS components with explained variance in brackets of the data of identified VOCs from non-infected (CON) and CMV infected pepper plants. (**a**) Score plot of the first two PLS components with explained variance in brackets. The ellipse defines Hotelling’s T^2^ confidence region (95%) and (**b**) Loading plot of the PLS-DA components that shows the contribution of each of the compounds to the first two principal components. For the interpretation of numbers refer to Table 2.

**Figure 5:**
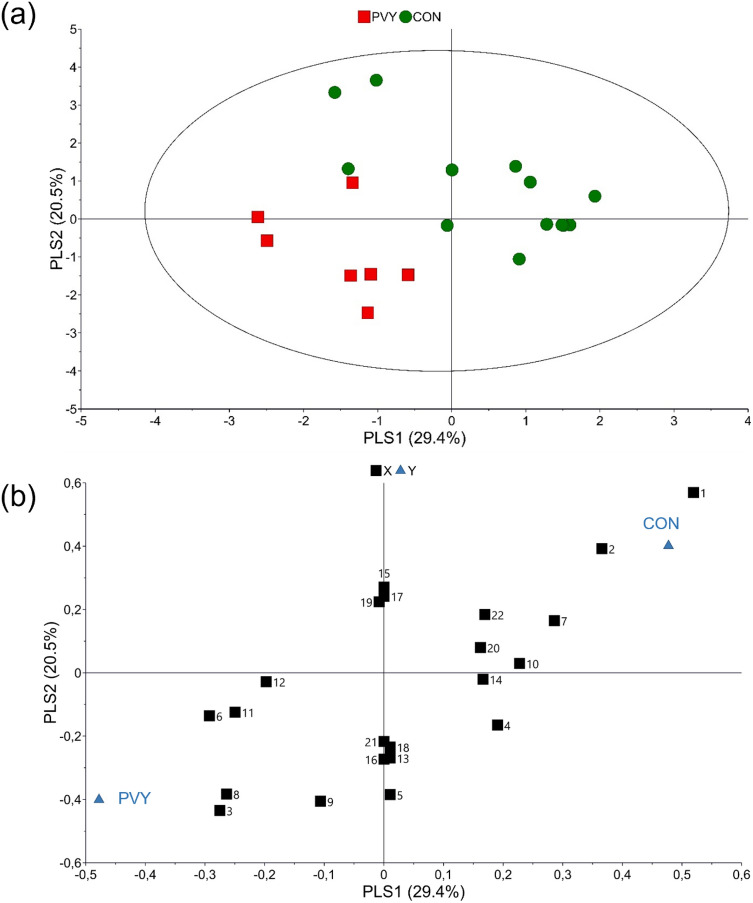
Projection Latent Structures Discriminant Analysis (PLS-DA) of the first two PLS components with explained variance in brackets of the data of identified VOCs from non-infected (CON) and PVY infected pepper plants. (**a**) Score plot of the first two PLS components with explained variance in brackets. The ellipse defines Hotelling’s T^2^ confidence region (95%) and (**b**) Loading plot of the PLS-DA components that shows the contribution of each of the compounds to the first two principal components. For the interpretation of numbers refer to Table 3.

In total 66 VOCs have been identified from non-infected and virus-infected pepper plants (Supporting Information Table S1). The VOCs profiles of CMV and PVY infected plants differ quantitatively and qualitatively to those of non-infected plants (Fig. 2; Supporting information Table S1, Figs. S1, S2). There is a tendency for lower emission of VOCs in virus infected pepper plants but that was not found to be statistically significant (χ^2^ = 5.671, df = 2, *p* = 0.059). In CMV-infected pepper plants 18 VOCs were identified that were not detected in non-infected and PVY-infected pepper plants. CMV-infected pepper plants emitted hexanal, terpenes (*α*-pinene, limonene), sesquiterpenes (*β*-elemene, *β*-longipinene) and homoterpene (*Ε*)-4,8,12- trimethyl-1,3,7,11-tridecatetraene [(*Ε*)-ΤΜΤΤ] which were absent from non-infected and PVY-infected pepper plants. In non-infected pepper plants, 6 VOCs have been identified that were not found in virus-infected pepper plants. The chemometric analysis showed that virus-infected and non-infected pepper plants are separated based on volatiles emitted (Fig. 3a). The first two principal components explained 41.9% and 15.6% of the variance, respectively. According to the loading plot, the first principal component separates CMV paper plants from the two other treatments, while second principal component clearly separates virus infested pepper plants from control ones (Fig. 3b). The model identified 21 compounds with variable importance for the projection (VIP) values > 1 (Table 1). Interestingly, the volatile emissions of 11 compounds out of the total 21 shown in Table S1 were significantly different among treatments. Virus infected pepper plants were mostly correlated with the emissions of ester 1, ester 2, ester 4, heptadecane, 2,6,10-trimethyl-dodecane and unknown 6. VIP compounds that characterize control pepper plants are 2,2,4,6,6-pentamethyl heptane, 2-ethyl-1-hexanol, alkane 3, undecane, alkane 10, dodecane and 2,4-dimethyl heptane. Further pairwise PLS-DA models between the blends emitted by plants infested by CMV and the control plants and PVY versus control plants were carried out. PLS-DA analysis yielded a separation between CMV-infested and control plants (Fig. 4). In total, 24 compounds contributed most to the separation (Table 2). Terpenes (limonene and *α*-pinene) and sesquiterpene *β*-elemene, that were detected only in CMV-infected pepper plants, were positively correlated to CMV-infected pepper plants. Figure 5 shows the separation between PVY-infected plants and control pepper plants. In this case, 22 compounds had a VIP value higher than 1 (Table 3). Ester 4, 2,6,10-trimethyl-dodecane, heptadecane, alkane 4, ester 2 and ester 1 were influenced by PVY-infection. These compounds found in higher levels also in the headspace pf PVY-infected plants (Table S1).

**Table 1:** Values of variable importance to the projection (VIP) of volatiles.

No	Compound	VIP value
1	Ester 2	2.51
2	2,2,4,6,6- pentamethyl heptane	2.50
3	Heptadecane	2.31
4	2-ethyl-1-hexanol	2.14
5	Ester 1	1.99
6	Unknown 6	1.89
7	Pentadecane	1.84
8	Ester 4	1.83
9	Tridecane	1.70
10	Alkane 10	1.69
11	2,6,10-trimethyl-dodecane	1.66
12	Alkane 3	1.62
13	2,4-dimethyl heptane	1.56
14	Alkane 8	1.32
15	Undecane	1.31
16	Camphor	1.28
17	Dodecane	1.20
18	Alkane 9	1.15
19	Camphene	1.12
20	Alkane 4	1.10
21	Octadecane	1.09

**Table 2:** Values of variable importance to the projection (VIP) of volatiles for PLS-DA _CMV-CON_.

No	Compound	VIP value
1	Alkane 10	2.18
2	2-ethyl-1-hexanol	2.13
3	Ester 2	2.11
4	Pentadecane	2.07
5	Tridecane	2.03
6	2,4-dimethyl heptane	1.92
7	*β*-elemene	1.63
8	Camphor	1.53
9	Dodecane	1.51
10	Tetradecane	1.46
11	Alkane 3	1.43
12	Alkane 9	1.41
13	Ester 1	1.36
14	Limonene	1.26
15	Alkane 8	1.21
16	Camphene	1.20
17	4-methyl dodecane	1.20
18	Undecane	1.17
19	2,6,10-trimethyl-dodecane	1.16
20	2,2,4,6,6- pentamethyl heptane	1.12
21	Alkane 12	1.12
22	Alkane 11	1.11
23	α-pinene	1.06
24	Ester 4	1.06

**Table 3:** Values of variable importance to the projection (VIP) of volatiles for PLS-DA _PVY-CON_.

No	Compound	VIP value
1	2,2,4,6,6- pentamethyl heptane	3.22
2	2-ethyl-1-hexanol	2.25
3	Ester 4	2.00
4	Undecane	1.96
5	Alkane 2	1.90
6	Heptadecane	1.87
7	Alkane 10	1.80
8	2,6,10-trimethyl-dodecane	1.72
9	Alkane 4	1.68
10	Alkane 3	1.59
11	Ester 2	1.59
12	Ester 1	1.40
13	Alkane 11	1.37
14	Tridecane	1.31
15	Octadecane	1.20
16	Alkane 5	1.18
17	p-xylene	1.18
18	Alkane 12	1.10
19	Hexadecane	1.07
20	4-methyl octane	1.05
21	Alkane 9	1.04
22	Tetradecane	1.03

## Discussion

Plants respond to insect herbivory by enhancing their defense mechanisms either directly or indirectly^42^. Directly, by producing plants toxins, digestion inhibitors and herbivore-induced plant volatiles (HIPVs) repellent herbivorous^17,43^; indirectly by emitting HIPVs that attract natural enemies of herbivores^44,45^. Parasitoids benefit by this interaction by increasing their foraging efficiency and parasitization success^46^, although the opposite is not excluded^47^. Vector-transmitted pathogens have been proven to alter the volatile profile of host plants to attract vectors and consequently enhancing pathogen dispersal and proliferation^11,13^. However, this is a species specific interaction as in other plant virus combinations no attraction of aphids to virus infected plants was observed^48^. Previously, attraction of parasitoids to virus infected plants has been shown for the whitefly-vectored tomato yellow leaf curl virus (TYLCV)^49^. Attraction of parasitoids to plant pathogen induced volatiles has been shown also for the psyllid parasitoid *Tamarixia radiata*, that it was attracted to odors released by citrus trees infected by the bacterium *Candidatus* Liberibacter asiaticus (Las) which causes the citrus greening huanglongbing^30^. In the above studies parasitoid attraction to pathogen infected plants was achieved even without the presence of their herbivore hosts. In the pioneer study of Mauck et al.^34^, *A. colemani* parasitoids didn’t show any preference for the odors of CMV-infected plants or non-infected plants, but another plant species has been used, *Cucurbita pepo*, and plants were simultaneously infested by *M. persicae* aphids^34^. The presence of aphids might have obscured the effect of the virus as aphids themselves cause HIPVs that attract parasitoids^36,37^. Similarly, *T. radiata* parasitoids didn’t show any preference for bacterial infected plants when their psyllid hosts were present^30^. In our assays, *A. colemani* parasitoids were able to discriminate non-infected and virus-infected plants and were attracted to the odors of both CMV and PVY infected plants. In all cases plants were free from their host aphids. To our knowledge, this is the first study that demonstrates a parasitoid species attraction towards virus infected plants without the presence of aphids. Presence of aphids might have confounded the attraction of parasitoids to virus infected plants due to aphids own emitted VOCs^50^ or due to aphids induced HIPVS^35,36^. We cannot exclude an increased parasitoids’ attraction towards virus-infected plants if they bear the aphid hosts/vectors but nevertheless, our findings are relevant because it is likely that both viruses exist in nature without the presence of aphids as except of aphid transmission, they are either seed or mechanically transmitted^51,52^. The biological or evolutionary drives behind the observed behavior are still obscure as it is difficult to identify a clear advantage exclusively for each of the four parties involved. Thus, parasitoids may benefit by identifying virus infected plants through earlier location of a habitat that is more likely to harbor host plants with aphids and thus resulting in more efficient foraging. Plants may also benefit by attracting natural enemies as they will decrease herbivore abundance in the plant community and consequently virus spread and prevalence. Early arrival of parasitoids as a response to virus-induced volatiles would favor biological control of aphid pests. Successful early biological control of aphids has been associated with reduction of aphid-vectored plant virus as early presence of natural enemies deter establishment of aphid^53^. On the other hand, the attraction of parasitoids to virus infected plants might favor virus transmission as aphids could respond to parasitoid presence by dispersing and thus further facilitate the spread of the virus in the plant community^54^. Considering equivalent studies with virus insect vectors further research on the interaction of insect enemies and virus infected plants should take into account a number of factors; among others, lack of uniformity of host phenotypes developed after a virus infection, changes caused in plant physiology during virus infection progress, differences in the mode of virus transmission, which are related to the duration of probing and feeding which are required for virus vectors to acquire and inoculate distinct types of plant viruses.

Virus infection caused the emission of VOCs that are identified HIPVs^55–57^. Our results clearly showed that virus infection elicited emission of VOCs that are known to have a behavioral role in insect plant interactions both for herbivores and their natural enemies^55,58^. Plant viruses as well as insect herbivores elicit plant defence signal-transduction in the jasmonate (JA), salicylic acid (SA) and ethylene (ET) pathways^44,59^. Infection of plants by viruses interferes with the physiological SA and JA defense signaling by creating more favourable conditions for the aphid vectors as well their attraction to the infected plants^6,7^. In our study, CMV infection induced the emission of terpenoids and in some cases the reduction in emission rates of monoterpenoids (camphor, isoborneol). *Aphis gossypii* infestation induced emission of terpenoids among other VOCs in cotton and cucumber plants^60,61^. Although CMV infected *C. pepo* plants were found to be nutritionally inferior for *A. gossypii,* aphids that are attracted to virus-infected plants, might disperse rapidly facilitating the spread of the non-persistent virus^13^. We don’t know if CMV or PVY infected pepper plants, are inferior for *A. gossypii* too. In case they are and aphids are dispersing from the plants, parasitoids will be attracted to plants where it is less likely to find aphid hosts to parasitize, leaving a portion of the vector population free of parasitism resulting in further spread of the virus in the plant community^30^. However, this remains to be confirmed by further experimental tests.

Our results contribute to the understanding of the complex interaction of plants, pathogens insect vectors and their natural enemies. However, in natural environment insects encounter a much more complex array of volatiles that they need to utilize for making behavioral decisions. Therefore, further investigation by combining laboratory and semi-field/field studies is needed for understanding the plant–insect interactions and elucidating the role of the virus induced VOCs in the behavior of parasitoids. The identification of potential compounds that act either as attractants or repellents either for the parasitoids or the aphid vectors will facilitate proper pathogen management.

## Materials and methods

### Plants, viruses, and insects

Pepper plants (*Capsicum annuum* cv. Yolo Wonder) were used in all experiments. Plants were kept in an insect-proof greenhouse with controlled environmental conditions at the premises of Benaki Phytopathological Institute (BPI) (Kifissia, Greece). Greenhouse conditions were temperature typically at 25 °C/20 °C (day/night), and photoperiod adjusted to 16 h-light with supplementary to daylight illumination provided by GreenPower LED flowering DR/W lamps (22 µmol/s). Plants were grown in soil-less potting medium (Potgrond P, Klasmann) in pots with dimensions 90 mm × 90 mm × 100 mm. Plants were not subjected to any pesticide treatment.

Viral inocula were prepared from CMV- or PVY-infected *Nicotiana* sp. leaf tissues, ground in 10 mM-sodium phosphate, 0.2% w/v DIECA buffer pH 7, in a 1:3 w/v dilution, containing 3% w/v active carbon and carborudum. Plants at the third to fourth true leaf stage were rub- inoculated^62^ with CMV or PVY inoculum, onto the youngest fully expanded leaf. Inocula were also applied to local lesion hosts of CMV and PVY (*Chenopodium quinoa* and *C. amaranticolor,* respectively) to quantify the infectivity of each inoculum. Confirmation of infection for each virus was performed following plant use for behavioral experiments and volatile collections, using double-antibody sandwich enzyme-linked immunosorbent assay^63^ (DAS-ELISA) utilizing commercial antibodies (LOEWE Biochemica GmbH). Samples were considered positives when their A405 was higher than three times the mean A405 of three non-infected control samples. Mock inoculated (with buffer only) pepper plants were the healthy negative controls. Parasitoid treatments followed after 14 days of virus or mock inoculations. All methods were performed in accordance with the relevant guidelines, and national and EU regulations.

*Aphidius colemani* was reared on *Aphis fabae* maintained on *Vicia faba* plants. The parasitic wasp *A. colemani* was obtained from the commercial company Koppert Hellas. Pupae of the parasitoid were introduced into net rearing boxes (51 × 51 × 41 cm) (Bugdorm, Taiwan). Rearing boxes were kept in standardized conditions in a climate chamber (23 ± 2 °C, RH 75 ± 5% and photoperiod of 16:8 L:D) until adults emerged. Adults were provided with water and honey ad libitum and potted *V. faba* plants bearing *A. fabae* colonies. New *A. fabae* mummies were transferred to new rearing boxes for continues rearing. Newly emerged (up to 24 h old) parasitic wasps were collected daily between 9:00 and 11:00 AM and used in the experiments. Behavioral experiments were carried out in an insect climate chamber at 25 ± 2 °C, 75 ± 10% RH, and a photoperiod of 14:10 h (L:D). Only females were used in the experiments.

### Chemicals

The adsorbent material 80/100 mesh Porapak Q was supplied from Supelco (Supelco Inc. Bellefonte, USA). MS-grade methanol, diethyl ether and *n*-pentane were purchased from Fisher (Fisher Chemicals, Bishop, UK). The standard mixture of *n*-alkanes C_8_–C_20_ 40 mg L^-1^ for the calculation of the retention indices was purchased from Sigma-Aldrich (Stenheim, Germany).

### Olfactometer behavioral experiments

Attraction of adult female parasitoids to virus infected pepper plants was assessed by a Y-tube olfactometer. A combination of a pepper plant infected either by CMV or PVY versus clean air or non-infected (control) pepper plant was offered to the adult parasitoid. The responses were assessed in a glass Y-tube olfactometer with 1 cm internal diameter, 10 cm main arm length and side arms 8 cm long. Plants were introduced into a 10 L glass jar connected with Teflon tubing in each arm. For clean air, an empty jar was used. Air was pumped (Dymax 5, Charles Austen Pumps Ltd, UK) through an active charcoal filter and re-humidified by passing through a bottle with tap water before directed into each jar connected to the one of the two arms of the olfactometer. The olfactometer was lined underneath with filter paper and evenly lightened for uniform lighting. Air flow rate was approximately 60 mL min^−1^. For each bioassay, a single female *A. colemani* was introduced into the central arm of Y-tube and left for 5 min to make a choice. A choice was recorded when a female was crossing 2 cm within the side arm and stayed for 15 s. A wasp which did not make a choice within 5 min was recorded as a ‘no response’.

Tests were conducted from 10:00 to 14:00 h. In all bioassays, after three runs the test stimulus positions were reversed to avoid any directional bias. After three replicates, the olfactometer was thoroughly washed with soap and water and rinsed with acetone before oven-dried at 120 °C. For each odour combination a single plant was used for each experimental day. Six wasps were used per day and odour combination to form a replicate. At least 22 replicates were performed for each combination.

### Collection of volatiles

Collection of plant volatiles was performed in laboratory. Potted pepper plants 3 weeks old (2 weeks post inoculation for infected plants) were transferred from the greenhouse nursery to the laboratory. The pot of each plant was hermetically covered with aluminum foil to prevent interaction with VOCs from soil and roots. Subsequently, each plant was left for 30 min for acclimatization before being placed in a glass container (10 L). Plants with any sign of mechanical damage were discarded. VOCs collection was performed by dynamic headspace sampling^64^. Ambient air was purified through an activated charcoal filter made with glass tube (10 cm length × 1.5 cm i.d.) containing 0.5 g activated charcoal (Merck, Germany) tapped with glass wool (extra fine, Assistent, Germany) and passed through the glass container by using a Dymax 5 vacuum pump (Charles Austen Pumps Ltd., UK) set at 360 mL min^−1^ flow rate. Plant volatiles were adsorbed onto a Teflon made trap (5 cm length × 4 mm i.d.) containing 75 mg Porapak Q, tapped with a 2 mm glass wool and 3 mm Teflon tubes on each end. The collection period was 6 h. After the collection, the adsorbent traps were eluted immediately with 500 μL of *n*-pentane. The eluates were stored in a freezer (− 20 °C) in a sealed vial until chemical analysis. Before analysis, the samples were concentrated to 100 μL under a gentle stream of nitrogen. In total, 6 pepper plants infected by CMV, 7 pepper plants infected by PVY and 13 non-infected pepper plants (controls) were used for VOCs collection.

### Identification

Identification of volatiles from headspace extracts was performed using gas chromatography-mass spectrometry (GC–MS). One microliter of the extract was used for the analysis. It was injected in a Varian CP-3800 GC, with a 1079 injector coupled with a 1200L quadrupole mass spectrometer. Separation of the analytes was performed with a TG-5MS capillary column (5% diphenyl/95% dimethyl polysiloxane) with dimensions 30 m length, 0.25 mm i.d., 0.25 μm film thickness (Thermo Scientific, Waltham, USA). Spitless mode was set for 1 min. The flow rate of the carrier gas helium was 1 mL min^−1^. The oven temperature was maintained at 50 °C for 5 min, increased with a rate of 3 °C min^−1^ to 170 °C and with a rate at 20 °C min^−1^ to the final temperature of 250 °C. Mass spectrometer was operated in Electron ionization mode (EI) with ion energy of − 70 eV, filament current 50 μA and source temperature 200 °C. Data acquisition was performed in full scan (MS) with scanning range 40–300 amu. Tentative identification was achieved by comparing their elution order, the mass spectra with those from mass spectra libraries (Adams 2007, NIST 2005, Wiley 275) and literature data^65,66^. We also used retention indices (RI) of a series of n-alkane (C_8_-C_20_), using the formula: 100n + 100 [(R_t_ (X) − R_t_ (N))/(R_t_ (N + 1) − R_t_ (N))], which is based on retention times of linear alkane standards; n = number of carbon atoms of the alkane N; R_t_ (X) = retention time of target compound; R_t_ (N) = retention time of N alkane which elutes before X; R_t_ (N + 1) = retention time of alkane eluting after X. Wherever possible, retention time and mass spectra were compared with commercial standards. The total ion chromatogram was processed by Varian MS Workstation software (version 6.9) based on the retention time and mass spectrum.

### Statistical analysis

To investigate whether parasitoid preference differed between the two combinations of virus-infected plants, data were analysed using logistic regression [i.e. a generalized linear model (GLM)] with a binomial distribution and a logit link function] with virus species as fixed factor. A quasi-binomial distribution was fitted in the model due to overdispersion. Since one plant was used for each experimental day and six wasps were used per day and odour combination to form a replicate, we used as response variable the number of wasps choosing the virus-infected plants out of the total number of responding wasps^67^. To determine under dual-choice conditions whether there was a significant preference for one of the offered plant treatments, we used (GLM) with a binomial distribution and a logit link function with virus treatment as fixed factor. The number of wasps choosing the virus-infected plants out of the total number of responding wasps was used as response variable. Data were analysed with SPSS. Non-responding individuals were excluded from statistical analyses.

Volatile compounds, measured as peak area and normalized with the peak area of internal standard, were log-transformed and processed by projections to latent structures-discriminant analysis (PLS-DA) using SIMCA 16.0 software (Umetrics, Umeå, Sweden). The Pareto scaling method was applied to the dataset before PLS-DA processing. Additionally, compounds with a variable importance for the projection (VIP) value higher than 1 were also generated. VIP values estimate the importance of each variable (compound) in the projection and is often used for variable selection^68^. Non-parametric Kruskal–Wallis (SPSS) was performed to identify differences in the quantities of the total VOCs amount and for each identified compound among different plant treatments.

## Supplementary Information

Supplementary Information.

## Data Availability

Most of the data generated or analysed during this study are included in this published article (and its Supplementary Information files). All other datasets generated during and/or analysed during the current study are available from the corresponding author on request.
