# Supplementary material for: Plant viruses induce plant volatiles that are detected by aphid parasitoids
Source: Sci Rep. 2023 May 30;13:8721. doi: 10.1038/s41598-023-35946-3 (PMC10229628; doi:10.1038/s41598-023-35946-3)
Supplement: Supplementary file 1 — Supplementary Information. [file 41598_2023_35946_MOESM1_ESM.docx]

**Supporting Information**

**PLANT VIRUSES INDUCE PLANT VOLATILES THAT ARE DETECTED BY APHID PARASITOIDS**

PANAGIOTIS MILONAS^1,*^, EIRINI ANASTASAKI^1^, AIKATERINI PSOMA^1^, GEORGIOS PARTSINEVELOS^1^, GEORGIOS FRAGOPOULOS^1^, OXANA KEKSTIDOU^2^, NIKON VASSILAKOS^2^ AND APOSTOLOS KAPRANAS^3^.

*^1^ Scientific Directorate of Entomology & Agricultural Zoology, Benaki Phytopathological Institute, 8 Stefanou Delta Street, 14561 Kifissia, Greece*

*^2^ Scientific Directorate of Phytopahtology, Benaki Phytopathological Institute, 8 Stefanou Delta Street, 14561 Kifissia, Greece*

*^3^ Laboratory of Applied Zoology and Parasitology, School of Agriculture, Aristotle University of Thessaloniki, 541 24, Thessaloniki, Greece*

* Corresponding author: Panagiotis Milonas. E-mail: [p.milonas@bpi.gr](mailto:p.milonas@bpi.gr)

The following table and figures are included in the supporting information:

**Table S1**. Relative response factor expressed as the ratio of the peak area of the sample to the peak area of the internal standard.

**Table S1.** Relative response factor expressed as the ratio of the peak area of the sample to the peak area of the internal standard.

|  |  |  | **Control** | | **CMV** | | **PVY** | |  |
| --- | --- | --- | --- | --- | --- | --- | --- | --- | --- |
| **RI _theor_^1^** | **RI  _exp_^2^** | **Compounds** | **Mean ± SE** | **n** | **Mean ± SE** | **n** | **Mean ± SE** | **n** | **Statistical characteristics** |
| *Monoterpenoids* | | |  |  |  |  |  |  |  |
| 932 | 938 | *α*-Pinene |  |  | 1.146±0.633 | 3 |  |  |  |
| 946 | 947 | Camphene | 0.136±0.030 | 8 |  |  |  |  |  |
| 1008 | 1006 | 3-*δ*-Carene |  |  | 1.016±0.205 | 6 |  |  |  |
| 1024 | 1024 | Limonene |  |  | 0.891±0.369 | 3 |  |  |  |
| 1083 | 1082 | Fenchone |  |  | 0.064±0.006 | 4 |  |  |  |
| 1135 | 1131 | *E*-Pinocarveol |  |  | 0.081±0.016 | 3 |  |  |  |
| 1141 | 1136 | Camphor | 2.276±0.661 | 6 | 0.390±0.113 | 3 |  |  | χ^2^=3.27, df=1, P=0.071 |
| 1140 | 1138 | *E*-Verbenol |  |  | 0.118±0.031 | 3 |  |  |  |
| 1155 | 1151 | Isoborneol | 0.139±0.053 | 7 | 0.077±0.003 | 3 |  |  | χ^2^=0.12, df=1, P=0.732 |
| 1158 | 1153 | *E*-Pinocamphone |  |  | 0.049±0.010 | 3 |  |  |  |
| 1160 | 1156 | Pinocarvone |  |  | 0.086±0.025 | 3 |  |  |  |
| 1167 | 1170 | Menthol |  |  | 0.064±0.020 | 3 |  |  |  |
| 1186 | 1186 | *α*-Terpineol |  |  | 0.102±0.040 | 3 |  |  |  |
| 1287 | 1281 | Bornyl acetate |  |  | 0.083±0.027 | 3 |  |  |  |
| 1298 | 1301 | Carvacrol |  |  |  |  | 0.447±0.068 | 3 |  |
| *Sesquiterpenoids* | | |  |  |  |  |  |  |  |
| 1828 | 1828 | Isopropyl myristate |  |  | 0.045±0.005 | 3 |  |  |  |
| 1389 | 1384 | *β*-Elemene |  |  | 0.199±0.073 | 5 |  |  |  |
| 1400 | 1397 | *β*-Longipinene |  |  | 0.662±0.243 | 3 |  |  |  |
| 1580 | 1576 | 4,8,12-Trimethyl-1,3,7,11-trideca tetraene (TMTT) |  |  | 0.150±0.069 | 5 |  |  |  |
| *Oxygenated aliphatic compounds* | | |  |  |  |  |  |  |  |
| 801 | 799 | Hexanal |  |  | 0.495±0.130 | 6 |  |  |  |
| 834 | 832 | 4-Hydroxy-4-methyl 2-pentanone | 1.030±0.304 | 7 |  |  | 1.439±0.642 | 6 | χ^2^=0.00, df=1, P=1.000 |
| **1026** | **1028** | **2-ethyl-1-hexanol ^3^** | **7.443±2.796** | **11** | **0.222±0.046** | **6** | **0.782±0.336** | **7** | **χ^2^ =13.72, df=2, P=0.001** |
| 1100 | 1105 | Nonanal |  |  | 0.292±0.174 | 3 | 0.170±0.064 | 3 | χ^2^=0.05, df=1, P=0.827 |
|  | 1183 | Alcohol 1 |  |  |  |  | 1.009±0.349 | 3 |  |
| 1322 | 1326 | 4*Z*-*tert*-Butylcyclohexyl acetate |  |  |  |  | 0.108±0.026 | 3 |  |
|  | **1341** | **Ester 1** | **1.096±0.283** | **9** | **0.483±0.125** | **6** | **1.840±0.427** | **7** | **χ^2^ =7.57, df=2, P=0.02** |
|  | **1363** | **Ester 2** | **1.139±0320** | **9** | **0.304±0.104** | **5** | **1.921±0.482** | **7** | ***χ*^2^ =6.11, df=2, P=0.047** |
|  | 1574 | Ester 3 |  |  |  |  | 0.284±0.054 | 3 |  |
|  | **1594** | **Ester 4** | **0.196±0.048** | **13** | **0.100±0.015** | **6** | **0.353±0.070** | **6** | **χ^2^=6.22, df=2, P=0.044** |
| *Aliphatic hydrocarbons* | | |  |  |  |  |  |  |  |
| 810 | 809 | 2,3,5-Trimethyl hexane | 0.262±0.063 | 6 |  |  |  |  |  |
| **822** | **816** | **2,4-Dimethyl heptane** | **4.138±0.965** | **13** | **0.824±0.290** | **5** | **2.226±0.181** | **7** | ***χ*^2^ =7.26, df=2, P=0.027** |
| 862 | 856 | 4-Methyl octane | 0.734±0.162 | 13 | 0.424±0.118 | 4 | 0.336±0.020 | 6 | χ^2^=1.14, df=2, P=0.565 |
| 988 | 987 | 2,2,4,6,6-Pentamethyl heptane | 1.192±0.232 | 12 | 1.195±0.596 | 5 | 0.158±0.040 | 4 | χ^2^=5.52, df=2, P=0.063 |
|  | 1046 | Alkane 1 | 0.190±0.049 | 8 |  |  |  |  |  |
|  | 1054 | Alkane 2 | 2.766±0.567 | 12 | 1.013±0.69 | 3 | 1.355±0.111 | 4 | χ^2^=3.32, df=2, P=0.191 |
|  | **1061** | **Alkane 3** | **0.865±0.142** | **12** | **0.296±0.090** | **4** | **0.415±0.095** | **5** | **χ^2^ =6.42, df=2, P=0.04** |
|  | 1093 | Alkane 4 | 0.356±0.098 | 11 |  |  | 0.675±0.406 | 3 | χ^2^=0.06, df=1, P=0.815 |
| 1100 | 1100 | Undecane | 1.231±0.232 | 12 | 0.460±0.171 | 4 | 0.569±0.168 | 5 | χ^2^=5.72, df=2, P=0.057 |
|  | 1106 | Alkane 5 | 0.490±0.078 | 11 | 0.287±0.016 | 3 |  |  | χ^2^=1.36, df=1, P=0.243 |
|  | 1113 | Alkane 6 | 0.236±0.053 | 6 |  |  |  |  |  |
|  | 1126 | Alkane 7 |  |  | 0.182±0.058 | 3 |  |  |  |
| 1200 | 1200 | Dodecane | 0.179±0.032 | 7 | 0.092±0.035 | 5 |  |  | χ^2^=2.91, df=1, P=0.088 |
|  | 1209 | Alkane 8 | 0.243±0.038 | 9 | 0.093±0.006 | 3 | 0.230±0.020 | 5 | χ^2^=5.77, df=2, P=0.056 |
|  | **1261** | **4-Methyl dodecane** | **0.213±0.042** | **7** | **0.096±0.020** | **4** |  |  | **χ^2^ =4.321, df=1, P=0.038** |
|  | **1270** | **Alkane 9** | **0.454±0.077** | **9** | **0.149±0.036** | **4** |  |  | **χ^2^ =4.02, df=1, P=0.045** |
|  | **1278** | **Alkane 10** | **1.558±0.243** | **10** | **0.305±0.113** | **5** | **0.691±0.187** | **4** | **χ^2^ =10.06, df=2, P=0.007** |
| **1300** | **1300** | **Tridecane** | **1.007±0.272** | **10** | **0.168±0.091** | **5** | **0.437±0.095** | **7** | **χ^2^ =6.72, df=2, P=0.035** |
|  | 1322 | Alkane 11 | 0.519±0.111 | 13 | 0.205±0.062 | 5 | 0.354±0.064 | 6 | χ^2^=3.41, df=2, P=0.182 |
| 1365 | 1367 | 2,6,10-Trimethyl-dodecane | 0.320±0.077 | 7 |  |  | 1.358±0.592 | 6 | χ^2^=2.47, df=1, P=0.116 |
| 1400 | 1400 | Tetradecane | 1.465±0.557 | 8 |  |  | 0.534±0.133 | 7 | χ^2^=2.63, df=1, P=0.105 |
|  | 1493 | Alkane 12 | 0.172±0.054 | 10 | 0.054±0.009 | 4 |  |  | χ^2^=0.73, df=2, P=0.694 |
| 1500 | 1500 | Pentadecane | 0.292±0.080 | 7 | 0.034±0.011 | 3 | 0.243±0.084 | 6 | χ^2^=5.90, df=2, P=0.052 |
| 1600 | 1600 | Hexadecane | 0.295±0.116 | 7 |  |  | 0.340±0.138 | 6 | χ^2^=0.18, df=1, P=0.668 |
| **1700** | **1700** | **Heptadecane** | **0.142±0.039** | **10** | **0.057±0.008** | **5** | **0.323±0.082** | **5** | **χ^2^ =7.73, df=2, P=0.021** |
| 1789 | 1785 | Octadecene |  |  | 0.187±0.048 | 3 |  |  |  |
| 1800 | 1800 | Octadecane |  |  | 0.058±0.008 | 3 | 0.163±0.048 | 6 | χ^2^=2.78, df=2, P=0.249 |
|  | 2335 | Polysaturated hydrocarbon | 0.957±0.128 | 9 |  |  | 1.590±0.553 | 7 | χ^2^=0.34, df=1, P=0.560 |
| *Aromatic compounds* | | |  |  |  |  |  |  |  |
| 862 | 862 | *p-*Xylene | 1.630±0.552 | 6 |  |  |  |  |  |
|  | 1044 | Diethyl benzene |  |  | 0.193±0.029 | 4 |  |  |  |
| *Other* | | |  |  |  |  |  |  |  |
| 1231 | 1224 | Cyclohexyl isothiocyanate |  |  |  |  | 0.398±0.093 | 5 |  |
| *Unknown* | | |  |  |  |  |  |  |  |
|  | 1077 | Unknown 1 | 0.145±0.020 | 6 |  |  |  |  |  |
|  | 1301 | Unknown 2 |  |  | 0.082±0.023 | 5 |  |  |  |
|  | 1334 | Unknown 3 |  |  |  |  | 0.229±0.040 | 3 |  |
|  | 1591 | Unknown 4 |  |  | 0.073±0.028 | 3 |  |  |  |
|  | 1653 | Unknown 5 |  |  |  |  | 0.274±0.070 | 3 |  |
|  | **1654** | **Unknown 6** |  |  | **0.054±0.012** | **3** | **0.303±0.034** | **3** | **χ^2^=7.47, df=2, P=0.024** |
|  | 1983 | Unknown 7 |  |  | 0.102±0.021 | 3 |  |  |  |

*^1^RI_thr_: obtained from Adams (2007), NIST database.*

*^2^RI_exp_: Retention Index were calculated relative to C_8_–_20_ n-alkanes on a column with 5% diphenyl/95% dimethyl polysiloxane stationary phase.*

*^3^Values in bold indicate significant difference (p<0.05)*

***Figure S1:*** *Individual relative VOC emissions of the major detected compounds (with relative abundance >1) expressed as mean ± SE, per treatment.*

***Figure S2:*** *Individual relative VOC emissions of the minor detected compounds (with relative abundance <1) expressed as mean ± SE, per treatment.*
